# Foreign Correspondence

**Published:** 1888-08

**Authors:** A. Lagorio

**Affiliations:** Chiavi, Italy


### FOREIGN CORRESPONDENCE.

### THE FESTIVAL OF THE EIGHT HUNDREDTH ANNIVERSARY OF THE UNIVERSITY OF BOLOGNA.

MR. EDITOR: Bologna, the great metropolis of Italian studies, has celebrated the eight hundredth anniversary of the foundation of her famous university in the presence of the royal family and of representatives of the learned of every nation. Never before was seen such a reunion of famous talent as that of the 12th of June, 1888, in Bologna. It is this circumstance which gives to the festival such universal interest. To make it such the rapid means of communication which we possess in our day have contributed in a special way, these being the result of many of the scientific studies which eight hundred years ago, in the long and historic night of human thought, Bologna had awakened and promoted, during which time Italy predominated over the world in science, letters, and arts, as she had done in arms during the Roman epoch.

How brilliantly shone the star of the Athenæum of Bologna where Irnerius, first, then Bulgar, Gregorius, Accurtius, and many other champions of free thought taught is told by the histories of the schools of Paris and Padua, and many others which later on rose to celebrity. From Hungary, from Germany, and from France pupils came to hear lectures at Bologna, although the means of conveyance were of the most meagre character, strikingly in contrast with the marvelous celerity of communication which brought to Bologna teachers and students from France, Germany, England, Spain, Switzerland, Roumania, Austria, Hungary, Greece, Belgium,

Russia, Portugal, and United States of America.

The University of Bologna is the most important one of Italy. It stands third in the number of its students in the list of twenty-one universities which Italy possesses, Naples having 4,083 students, Turin 2,102, and Bologna 1,338.

The exact date of the origin of Bologna's university is really unknown. It is supposed that Emperor Theodosius was the founder; some attribute this honor to St. Petronius, while others give the honor to Irnerius, who from the documents extant must be admitted to have been the oldest teacher. Although it is thought that the year 1088 is the exact date of her origin, it is said there are documents in existence which tend to prove that it existed many years before. Others fix the date in 1111. At any rate, the University of Bologna is the oldest in the world. The approximate dates of foundation of some of the celebrated universities of Europe are as follows:

Bologna, Italy, 1088 (?); Paris, France, 1230; Valencia, Spain, 1209; Naples, Italy, 1224; Padua, Italy, 1228; Rome, Italy, 1245; Oxford, England, 1249 (?); Cambridge, England, 1254 (?); Montpellier, France, 1284; Pisa, Italy, 1343; Prague, Bohemia, 1340; Pavia, Italy, 1360; Vienna, Austria, 1365; Heidelberg, Germany, 1386; Cambridge, United States, 1636; Berlin, Germany, 1810.

In those times there were no buildings which could be called a university. Each professor gave his lectures at his home, or in halls rented for the purpose. It was not until about the year 1562 that they taught in the present building, called the Archigymnasium, where all the receptions, addresses, etc., were held during the present festival. The University of Bologna at first taught only laws, as the school of Salerno taught at that time medicine and that of Paris theology. But soon the number of studies increased and teaching divided into four faculties, that of law, theology, medicine, and fine arts. The university gained fame which spread throughout the world. It is said that at one time the number of students was over 10,000. At the present time this university ranks as the most important one in Italy. It possesses fine and rich museums, elegant and well-supplied laboratories, and an eminent faculty composed of men highly esteemed at home and abroad.

The enthusiasm, joy, and cordiality which prevailed among all the students and representatives of every nation, the pomp and the impressive character of the festivities held a few days ago, my pen is incapable of describing. The presence of the royal family, always ready to participate in the joy and happiness of their people, raised the enthusiasm to the highest pitch. On the 9th of June the foreign students began to arrive. At the depot every train on arrival was met with loud cheers and hurrahs. On one side there was a committee of professors welcoming their guests; on the other, hundreds of students receiving their companions with ringing cheers, embracings, handshakings, and congratulations of every sort.

Bologna was unrecognizable; the streets were full of people and animated; every train brought thousands of persons. The hotels were crowded and the foreign representatives were lodged in private homes. Crowds of happy students were seen in every street and recognized by their caps. The law students had on blue caps; those of medicine, red caps; the mathematicians, green, and those of philosophy and letters, white. All these colors mixed together produced a most lively effect.

The reception ceremony at the university building was really solemn. The large court-yard was packed with students, the windows fronting it were filled with ladies. The first lines were occupied by the representatives of the Universities of Berlin, Leipsic, Gratz, Athens, and Edinburgh. The rector of the latter university wore an elegant robe, and he was loudly cheered. The president of the students' committee, Mr. Pietri, spoke first. He welcomed all the companions present from so many parts of the civilized world. A representative from Athens University responded in the French language, saying that the respect of the Greeks for the *Alma Mater Studiorum* was great ; then followed a representative from Leipsic dressed in a rich and showy robe ; two other students stood at his side. One of them spoke in Italian, saying that the scientific world had sent their representatives to Bologna to make their contribution to the great occasion. A representative from Berlin spoke in a similar strain and was loudly cheered when he said that Germany loves Italy and wants to be her companion in adversity as well as in prosperity. Representatives from Gratz, Rome, Paris, etc., spoke in a similar happy manner and were all greeted with applause. This ceremony ended, they all went to Brunetti's theatre to hear the grand welcome oration delivered by Professor Pauzacchi. The humorous part of the day was the arrival of Padua's students with a mastodonic ox with gilt horns and covered with rich drapery. The Turin students brought a large barrel of good wine, with a student on top of it representing Bacchus, the god of wine; two other students represented a bacchant and a satyr. Next followed the students of Pavia with a phenomenal Parmesan cheese with a student on top representing the goddess Ceres. The procession was a mile long, and as all students were dressed in rich costumes the sight was indeed bewildering.

On the 10th there was a reception by the royal family from Rome, and the unveiling of a grand monument, dedicated to Victor Emanuel, occurred. In the evening there was a gala night at the theatre.

But the 12th of June will be remembered as the most glorious day that Bologna has ever witnessed. It was the greatest day of the festival. From early morning the professors began to meet at the university building. Their different costumes and languages blended in the common brotherhood of science. All were dressed in their rich college robes. The whole city was grandly decorated, and the streets were packed with people eager to witness the grand procession, which began to move about 9.30 a. m. First came the local associations with flags, followed by the mace-bearers of the University of Bologna carrying the old and artistic silver maces. Then came the students of Bologna with their colored caps, followed by the students of the Italian and foreign universities with their respective flags.

The Italian professors came next, dressed in black robes with ermine and wore the doctoral cap ; they were accompanied by their respective mace-bearers dressed in black with short pants and carrying rich silver maces.

The professors of foreign universities followed. First, came those from the United States. The New York professors made a grand impression, being dressed in rich dark-red robes, with impressive caps on their heads. The professors of Baltimore, Cambridge, Iowa, Ithaca, Cornell, New Haven, Philadelphia, Michigan, Virginia, and Wisconsin were loudly cheered. South America had representatives from Buenos Ayres and Santiago.

Professor Raymond West represented the old University of Bombay, India, and was dressed in a damask robe, ornamented on the chest and shoulders with silver bands.

Australia had a professor from the University of Adelaide, and New Zealand had one from Wellington.

Then came the representatives from the European universities, as follows :

First, the professors from Austria-Hungary. Professor Abta, rector of the University of Kolosvar, was dressed in a rich Hungarian costume, and wore around his neck a chain ornamented with precious stones.

Then followed the professors of Cracow with large robes of black velvet ; then those of Buda-Pesth, Czernovitz, Gratz, Innsbruck, and Lemberg, all wearing black gowns. Those of Prague and Vienna wore blue velvet.

The professors of Brussels, Gand, and Liege wore black robes.

Then came the professors of Copenhagen.

France was represented by a strong body of doctors dressed in elegant violet, red, yellow, and orange colored robes with caps of the same color. Paris, Aix, Bordeaux, Caen, Lille, and Lyons were represented.

Germany came next with a great variety of costumes. The Professors of Berlin had on black robes with red collars and caps of the same color. Those of Bonn, Breslau, Erlangen, Göttingen, and Greifswald wore black robes with mantles of red velvet richly ornamented, which made them look like cardinals.

The doctors of Halle, Heidelberg, Jena, Kiel, Königsberg, and Leipsic were dressed in dark red; those of Marburg had on black robes with a large mantle of blue and violet colors richly embroidered. Lastly, came those of Munich, Rostock, Strasburg, Tübingen, and Würzburg.

Then came the representatives of Athens, Greece.

The professors of England, Ireland, and Scotland followed; those of the Universities of London, Aberdeen, Cambridge, Durham, Glasgow, Manchester, Oxford, and St. Andrew's wore large robes of red color with white collars; those of Dublin, Galway, and Edinburgh had on violet robes and blue collars.

Holland had professors from Amsterdam, Leyden, Utrecht, and Groningen. Portugal had representatives from the University of Coimbra, and Roumania from those of Bucharest and Jassy.

Russia was represented by professors from the Universities of Dorpat, Helsingfors, Kasau, and St. Petersburg.

Spain by professors from Madrid, of Granada, and Orviedo in black gowns with scapularies, which made them look like so many monks; some had a red scapulary, others violet; they carried rich gold chains and wore caps covered with falling fringes.

Sweden and Norway had professors from Christiania, Lund, and Upsala; one carried a little sabre at the side and a pointed cap; and the other a silk hat made up in little folds.

Lastly, came the professors from Switzerland—the Universities of Bâsle, Berne, Geneva, and Zurich.

Next came the great flag of the University of Bologna, followed by all the professors of this university.

This fantastic procession, after passing through the principal streets, amid cheers from every side, proceeded to the Archigymnasium. Every professor was presented with a little branch of laurel and oak. The large court-yard of this historic Archigymnasium was covered by a great pavilion ornamented in red and white. In the centre there was the coat-of-arms of Bologna, with the motto, *Resonavit fama per orbem.* The throne was placed under a large red-velvet pavilion. At the right of the throne was placed the city flag, with the motto, *Libertas;* to the left the banner of the university, with the motto, *Alma Mater Studiorum.* At ten o'clock the procession entered the court-yard, and all took their respective places. The royal family also entered and took seats on the throne, the king being dressed in a grand uniform of a general. A song, composed expressly for the occasion, was sung by a chorus of voices, after which the rector of the university, Professor Coppellivi, made a short address of thanks. Honorable Baselli, Minister of Public Instruction, then delivered an oration, during which he was frequently interrupted by applause. The poet, Professor Joshua Carducci, was next introduced, and read an address and spoke in glowing terms of the past and present studies of the University of Bologna.

The foreign representatives spoke next. Professor Vogel, of Vienna, speaking in

Italian, hailed Bologna as always keeping lighted the lamps of knowledge. He presented as an homage a parchment and deposited it in a basket placed before the rector.

Klinger, professor of theology in Buda-Pesth, speaking in Latin, presented the good wishes of Hungary, and concluded by saying, "May the School of Bologna long live and prosper."

Pictak, of Lemberg, presented some books.

Rivier, of Belgium, spoke in French and in behalf of his country, and congratulated Bologna on her having the oldest university. He presented some books and parchments.

Professor Scharling, in behalf of the Danish doctors, presented some books.

Professor Hamilton, of Lund, spoke for Sweden and Norway. Speaking in Italian, he said : "At one time they said, '*Ex oriente lux*,' but the Scandinavians say, '*Ex Italia lux*.' Mountains and seas divide us, but virtually we are connected by science, truth, and liberty." He also offered parchments.

Nation after nation followed. When each name was called out the enthusiasm increased in an astounding manner.

When the French doctors came forward dressed in their splendid robes the applause was deafening. Professor Bufnoir, of Paris, mentioned, in an eloquent manner, the relations existing between the universities of Paris and Bologna. They deposited rich parchments.

Professor Hoffman, of Berlin, spoke in Italian and said that half a century ago he was a student in Bologna. He hailed Bologna as the *alma mater* of studies. All the German professors presented rich parchments and books.

Professor Arethens, of Athens, Greece; Professor Jebb, of Cambridge, England; Vasconcellos, of Coimbra, Portugal; Professor Jonesc, of Jassy, Roumania; Professor Wesselofsky, of St. Petersburg, Russia; Piera, of Madrid, Spain; Vogt, of Geneva, Switzerland; Vanzim, of South America all made addresses and presented books and parchments.

Professor Stickney, of Hartford, United States, made a fine address and was loudly cheered.

Lastly, Professor Gandien, of Bologna, made a speech in Latin. He saluted all the guests and wished prosperity to every nation. Amid cheers and applause the ceremony ended at two o'clock.

In the evening over 700 students were seated at a banquet. Telegrams were received from Christiania, Stockholm, and other places. Toasts were given in all languages. President Pietri hailed the companions of all nations, and especially those of Athens, the mother of arts and science. He hoped that France, Germany, England, Spain, and all other nations would work for the international federation of the people. He said that America teaches the world and that Europe ought to imitate the United States. These last remarks were greeted by a tremendous applause. Gladness and good feeling prevailed on all sides.

Over 400 professors of foreign and local universities were also entertained at a banquet offered by the government at another place. Toasts were also made in all languages hailing the king, Bologna, Italy, and all nations.

On June 13, the professors and students met at 9 o'clock a. m., at the Archigymnasium for the distribution of the honorary diplomas to the eminent foreign scientists. The royal family entered at ten o'clock and the ceremony commenced. The square was packed with an elegant and select audience. The sight was impressive. The band having played a selected piece of music composed expressly for the occasion, Professor Pelliccioni, a Greek scholar of reputation, made an address in Latin to the graduates. The presidents of the different faculties called the names of their respective graduates. First, came the philologists, then the mathematicians, the jurists, and lastly the doctors of medicine. The rector of Bologna's university presented to each one the diploma, and the doctoral ring of the athenæum, into which each graduate introduced a finger and immediately withdrew it.

Lastly, Professor Ceneri, a prominent legal gentleman, in a brilliant address, saluted the scientists present. He was congratulated by the king for his able speech, and warmly cheered by the vast audience.

At 1.30 p. m. over thirty associations, preceded by bands of music and flags, moved in line through the principal streets and entered the theatre to hear the address of Professor Giovanni Bovio, on the life of Giordano Bruno.

The prettiest episode of this memorable festival occurred on this day at the concert hall of the exposition building—the distribution of presents made by the Bologna ladies to the students. The ladies numbered at least 500, and the students pres- were not less than 2,000. The students on their part offered flowers to the ladies. A true festival of courtesy.

June 14 was dedicated to the commemoration of Galvani, which was held at the Archigymnasium in the presence of the foreign and local invited guests. Professor Albertoni made the address. He said that it was a happy thought to commemorate the centenary of Galvani with the centenary of Bologna's athenæum, by commemorating and remembering those men that made it famous. Galvani, more than any other scientist, excited the attention of the world, because with his discovery it was thought that the vital force was found. He demonstrated experimentally that the brain is the seat of intelligence. He mentioned the experiments and works of Galvani. He compared the teaching of those times with the present. He spoke of the modern progress of physiology. He alluded to many unsolved problems and urged the present generation to solve them, concluding that the best recompense of work is the eternal memory that history consecrates to them.

After this address the foreign and Italian representatives were presented with special medals commemorating the celebration of the centenary of Bologna's athenæum. The committee then proceeded to place crowns of laurel on the monument, on the house, and on the tomb of the great and immortal scientist.

Thus ended this remarkable festival, which has been solemn in every way, and a true declaration of the gratitude of every nation to the University of Bologna: *mater alma studiorum.* With this commemoration Italy has intended to confirm her past glories to the world, glories of science, of letters, and of arts; and it is right that while in her full triumph, with her new capital, Rome, she affirms to the world her power and greatness. A. LAGORIO.

CHIAVI, ITALY.

